# Circulating angiogenic factors in a pregnant woman on intensive hemodialysis: a case report

**DOI:** 10.1186/s40697-016-0096-7

**Published:** 2016-02-23

**Authors:** Ayub Akbari, Michelle Hladunewich, Kevin Burns, Felipe Moretti, Rima Abou Arkoub, Pierre Brown, Swapnil Hiremath

**Affiliations:** Division of Nephrology, Department of Medicine, University of Ottawa, Ottawa, Ontario Canada; Kidney Research Centre, Ottawa Hospital Research Institute, Ottawa, Ontario Canada; Clinical Epidemiology Program, Ottawa Hospital Research Institute, Ottawa, Ontario Canada; Department of Medicine, Division of Nephrology, Sunnybrook Health Sciences Centre, University of Toronto, Toronto, Ontario Canada; Department of Obstetrics and Gynecology, University of Ottawa, Ottawa, Ontario Canada; The Ottawa Hospital, Riverside Campus, 1967 Riverside Drive, Ottawa, ON K1H 7W9 Canada

**Keywords:** Pregnancy, Soluble fms-like tyrosine kinase, Placental growth factor, Chronic kidney disease, Dialysis

## Abstract

**Background:**

Pregnancy in patients on chronic hemodialysis therapy, though unlikely, does happen rarely. Intensive hemodialysis is thought to offer a better survival advantage to the unborn child. Circulating angiogenic factors are helpful for prognostication of pregnant patients with chronic kidney disease who are not on dialysis. Data on their utilization in dialysis patients, however, are limited.

**Case Presentation:**

We report the case of a patient with a history of interstitial nephritis who had a kidney transplant that failed after 8 years due to membranous nephropathy. She was initiated on hemodialysis three sessions per week and conceived after being on dialysis for 6 weeks. She was switched to intensive hemodialysis at 8 weeks of gestation and had a C-section because of hypertension at 35 weeks, with delivery of a healthy girl weighing 2012 g. Serum angiogenic factors (placental growth factor and soluble fms-like tyrosine kinase) were measured at 32, 33, and 34 weeks of gestation and at 1, 2, and 3 weeks postpartum. Serum angiogenic factors were similar to what has been reported for patients with chronic kidney disease and were not consistent with preeclampsia.

**Conclusions:**

Our case report expands on the literature regarding intensive hemodialysis and angiogenic factor utilization in pregnant dialysis patients. Our case report suggests that starting intensive dialysis early in pregnancy is safe and concentration of angiogenic factors are similar to those reported for patients without kidney disease, except for PIGF levels, which are somewhat higher.

## ABRÉGÉ

Bien que peu probable, une grossesse chez les patientes sous dialyse chronique survient en de rares occasions. Dans ces cas très précis, l’hémodialyse intensive est considérée comme le traitement offrant les meilleures chances de survie pour l’enfant à naitre. Chez les patientes enceintes souffrant d’insuffisance rénale chronique, mais non dialysée, les facteurs antiangiogéniques circulants améliorent le pronostic de la grossesse. Les données sont toutefois limitées en ce qui concerne le cas de patientes enceintes sous dialyse. Nous discutons du cas d’une patiente avec un historique de néphrite interstitielle et dont la transplantation rénale a échoué après 8 ans en raison d’une glomérulite extra-membraneuse. Elle avait à ce moment entrepris une dialyse à raison de trois séances par semaine. La patiente est tombée enceinte six semaines après le début du traitement par dialyse et a dès lors été transférée au traitement par l’hémodialyse intensive. Le bébé, une fille de 2 012 kg en parfaite santé, est né par césarienne à 35 semaines de gestation, car la mère souffrait d’hypertension. Deux facteurs antiangiogéniques ; le facteur de croissance placentaire *PIGF* ainsi que la tyrosine kinase *sFlt-1*, ont été dosés à 32, 33 et 34 semaines de gestation de même qu’une, deux et trois semaines postpartum. Les taux mesurés se situaient à des niveaux attendus chez les patients souffrant d’insuffisance rénale chronique et n’indiquaient pas de prééclampsie. L’étude de ce cas particulier vient enrichir la documentation existante en regard de l’utilisation de l’hémodialyse intensive chez les patientes enceintes. Ce cas précis laisse croire qu’il est sécuritaire pour l’enfant à naitre d’amorcer une telle procédure dès les premières semaines de la grossesse chez une femme souffrant d’insuffisance rénale chronique. Ce cas montre également que dans ces conditions, les concentrations sériques des facteurs antiangiogéniques s’avèrent similaires à celles rapportées pour une femme enceinte ne souffrant d’aucune néphropathie, à l’exception du PIGF dont la concentration est sensiblement plus élevée chez la patiente dialysée.

## Background

Pregnancy in patients with end-stage renal disease (ESRD) on dialysis therapy is uncommon. The reported conception rate is between 1 and 7.9 % for women of child-bearing age, but recent data indicate improvement in conception rates as centres have intensified dialysis regimens [1]. Patients who start dialysis during pregnancy have a more favorable outcome than those who become pregnant on dialysis, presumably due to the presence of residual renal function. Furthermore, there is an association between the hours of hemodialysis received and the chances of a live birth [2]. Conventional hemodialysis three times a week for 4 h in pregnant women is associated with much poorer outcomes compared to intensive hemodialysis (greater than 36 h per week) [2]. Still, the major morbidity and mortality for patients with chronic kidney disease (CKD) who are pregnant is secondary to preeclampsia (PE) [3]. PE is a placental disease which occurs in up to 5 % of all pregnancies, but rates are significantly higher in women with CKD [4, 5].

In the last decade, angiogenic factors such as placental growth factor (PIGF) and soluble fms-like tyrosine kinase (s-FLT-1) were found to be abnormal in the maternal circulation in women who developed PE [6]. Emerging data suggest that serial measurements of circulating angiogenic factors may help predict PE and adverse pregnancy outcomes [3, 7–10]. However, the data on angiogenic factors in pregnant patients on hemodialysis are limited [9, 11]. We measured PIGF and s-FLT-1 in a pregnant patient with end-stage renal disease secondary to interstitial nephritis whose renal transplant failed because of de novo membranous nephropathy, and she was started on hemodialysis therapy.

## Case presentation

A 33-year-old female with a history of ESRD secondary to interstitial nephritis had a preemptive kidney transplant which failed after 8 years, secondary to biopsy-proven de novo membranous nephropathy. She had had a history of unprovoked pulmonary embolism 4 years after the renal transplant and had been treated with oral anticoagulation for 2 years. The hematology consultant found no evidence of thrombophilia. Her cyclosporine dose had been tapered off within 1 month of starting dialysis. Her prednisone in a dose of 5 mg orally per day was continued throughout pregnancy. She was found to be 6 weeks pregnant after being on dialysis for 3 months. This was an unplanned pregnancy. The patient had a history of smoking but quit on learning of her pregnancy. She was switched to intensive hemodialysis (six dialysis sessions per week for a total dialysis time of 45 h per week) at 8 weeks of gestation. She received heparin 500 units i.v. per hour on dialysis to prevent dialyzer clotting (dialyzer utilized was FX 800 Fresenius Medical Care dialyzer), and tinzaparin for prophylaxis against pulmonary embolism. Blood flow was kept at 300 ml/min during dialysis, and dialysate flow was 500 ml/min. Dialysate potassium concentration was 3 mmol/l, bicarbonate concentration was kept at 25 mmol/l, and calcium concentration was kept at 1.75 mmol/L. Intentionally, no dietary restrictions were implemented. Her prenatal vitamin dose was doubled. She was also given oral zinc 15 mg per day. Blood work was monitored initially once weekly and then at least once every 2 weeks. Sodium phosphate was added to her dialysate at a concentration of 0.9 mmol/l at 12 weeks of gestation. She required increasing doses of erythropoietin from 8 weeks of gestation and increasing doses of intravenous iron from 12 weeks of gestation to maintain her hemoglobin above 90 g/l.

**Fig. 1:**
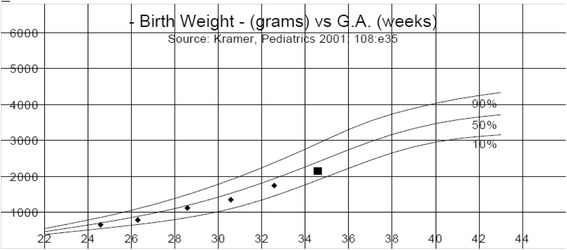
Estimated fetal weight by antenatal ultrasound demonstrated normal growth curve

In the first trimester, the patient developed symptoms of gastritis and was started on oral pantoprazole. She had an integrated prenatal screen (IPS) for aneuploidy which was negative as well as alpha-fetoprotein and pregnancy-associated plasma protein A was normal. At 19 weeks and 4 days of gestation, no structural fetal anomaly was detected by ultrasound. On uterine artery Doppler, there was no notching or abnormal pulsatility index or abnormal resistive index, signs which are helpful in predicting PE. The fetus had a normal growth curve throughout the pregnancy (Fig. 1) and normal umbilical artery Dopplers.

Blood pressure started to increase at 28 weeks gestation. She was started on labetalol 100 mg orally twice per day for systolic blood pressure in the range of 150 mmHg and diastolic blood pressure in the range of 100 mmHg, with good control until 33 weeks of gestation when blood pressure rose again, and the labetalol dose was progressively increased to 200 mg every 6 h. On clinical exam, she did not have evidence of volume overload.

Because of uncontrolled blood pressure (blood pressure 160/110 to 170/102) she underwent C-section at 35 weeks of gestation, and gave birth to a healthy girl weighing 2012 g. She was not treated with magnesium sulphate. Liver function tests and platelet counts remained within the normal range throughout the peri-partum period. Placental pathology revealed a normal maternal vascular pattern. Placental weight at delivery was 306 g (expected 352 to 516 g).

Serum PIGF and s-FLT-1 were measured at 32, 33, and 34 weeks of pregnancy and at 1, 2, and 3 weeks postpartum. Blood was drawn immediately prior to the hemodialysis treatment. Both sFLT-1 and PIGF were measured by enzyme-linked immunosorbent assay (ELISA). The kit used for PIGF was Human ELISA Kit from Abcam®, Toronto, and for sFlt-1 was Human ELISA Kit from R&D Systems®, Minneapolis, MN.

All assays were performed in duplicate, according to the manufacturer’s instructions. The results are shown in Table 1. Serum sFLT-1 remained within the normal range reported for patients without kidney disease, and PIGF levels were higher than the normal range [12] arguing against development of preeclampsia.

**Table 1:** Values of placental growth factor (PIGF) and soluble fms-like tyrosine kinase (s-FLT-1) and their ratio

	PIGF (pg/ml)	s-Flt-1 (pg/ml)	sFLT-1/PIGF
Gestational week 32	2809.2 (normal 54–1312)	1084 (normal 680–8042)	0.39 (normal 0.80–86.4)
Gestational week 33	1959.8 (normal 54–1312)	1142.5 (normal 680–8042)	0.58 (normal 0.80–86.4)
Gestational week 34	1535.9 (normal 43.6–1177)	1193 (833–11,643)	1.29 (normal 1.01–109)
Post-partum 1 week	774.6	68.5	0.09
Post-partum 2 weeks	667.2	149	0.22
Post-partum 3 weeks	728.5	117.5	0.16

### Discussion

We report a successful pregnancy in a patient who became pregnant after initiation of hemodialysis therapy. She was switched to intensive hemodialysis early in her pregnancy. The pregnancy was well tolerated, although her blood pressure became labile at 35 weeks of gestation. A C-section was performed and a healthy child was delivered weighing 2012 g.

Fertility is markedly reduced in patients with ESRD, and pregnancy in hemodialysis patients remains uncommon [13]. When pregnancy occurs, there is a high risk of mortality and morbidity to the mother and fetus. The outcome of such pregnancies depends on the dose of dialysis that the pregnant woman receives. Dialysis <20 h per week is associated with high mortality with live births ranging from 27 to 37 %. Infant survival has improved over the years with increasing dose of dialysis [13]. Dialysis of 17 ± 5 h is associated with an infant survival rate of approximately 50 % [2], but recent data suggest that higher doses of dialysis (greater than 36 h per week) are associated with decreased prematurity and improved infant survival [2]. Our patient underwent dialysis 45 h per week starting at 8 weeks gestation. Our case report therefore suggests that starting intensive dialysis early in pregnancy is safe, well tolerated, and may improve outcomes as previously reported [2]. The major complication of pregnancy on dialysis is the development of pregnancy-induced hypertension. A diagnosis of PE has important consequences on clinical management. The recommended management of PE is delivery of the baby. Indeed, if severe PE is diagnosed, the baby may need to be delivered prematurely to prevent maternal complications. A clinical diagnosis of PE is made during pregnancy in the presence of new onset hypertension and proteinuria after 20 weeks of gestation or in the absence of proteinuria; one of the following is required to make the diagnosis (a) decreased platelet count; (b) renal insufficiency; (c) abnormal liver function tests (d) pulmonary edema, and (e) central or visual symptoms [14]. In proteinuric patients with CKD, the diagnosis of PE is therefore challenging. In one series, patients with CKD who were suspected to have superimposed PE had kidney biopsy performed, and only 58 % had classic histologic evidence of PE [15].

Circulating angiogenic factors may play a pathogenic role in the development of PE [16]. These factors regulate placental development, including remodelling of the spiral arteries [17]. It is thought that in PE, faulty remodelling of spiral arteries leads to relative hypoxemia which increases production of sFlt-1. The increase in circulating sFlt-1 induces excessive binding of free PIGF leading to endothelial dysfunction, a hallmark of PE [16]. In the general population, high serum concentration of sFlt-1 and low levels of PIGF predict PE before development of clinical manifestations [16].

In patients with CKD, levels of circulating angiogenic factors represent promising markers to assist in the diagnosis of PE [9]. VEGF levels have been found to be similar in patients on hemodialysis as compared to healthy people [18]. The data on angiogenic factors in patients on established hemodialysis are limited, and indeed, our literature search uncovered only two case reports where circulating angiogenic factors were measured in pregnant women undergoing hemodialysis. Shan et al. [19] reported the case of a 34-year-old woman who conceived 1 month before initiation of hemodialysis and had to be delivered at 29 weeks and 2 days because of severe hypertension. No evidence of PE was found on pathological examination of the placenta. sFlt-1 levels were 2468 pg/ml, which is within the normal reference range. Shan et al. did not measure PIGF levels. Cornelis et al. [11] reported the case of a 21-year-old female who was started on intensive hemodialysis at 26 weeks of gestation. She was delivered at 35 weeks and 5 days of gestation because of sudden unexplained hypertension. sFLT-1 and PIGF levels were monitored from 21 weeks of gestation and remained in the normal range. Our patient was started on intensive hemodialysis at 8 weeks of gestation and also delivered because of hypertension at 35 weeks. Other than hypertension, the patient did not have any symptoms or signs of PE, lab parameters did not indicate the HELLP syndrome, and there was no intrauterine growth retardation (IUGR). Placental histology was unremarkable. The placental weight was low, perhaps related to the patient’s smoking status or poor vascular health with history of end-stage renal disease and history of kidney transplant with exposure to immunosuppressives. Circulating angiogenic factors were not consistent with a diagnosis of PE. In PE superimposed on CKD, the concentration of sFlt-1 is much higher (median, 13,519.5 pg/ml; range 6059–34,398 pg/ml) and PIGF levels are much lower (median, 32.6 pg/ml; range 55.9–2632 pg/ml), and the ratio of sFlt-1 to PIGF (which has a better diagnostic accuracy for preeclampsia [12]) is quite high (median, 435.79; range 160.9–1153.53) [9], compared to the results in our patient. Although our patient developed hypertension, the etiology is unlikely to be related to PE as a host of other factors might contribute to hypertension in a dialysis patient including sodium and volume excess, activation of the renin angiotensin system, altered endothelial function, and increased sympathetic activity. Thus, if we had access to the results of angiogenic factors in real time, premature delivery may have been avoided as there was no fetal indication for delivery and she was on a single antihypertensive agent and a second antihypertensive agent could have been added with close monitoring.

## Conclusions

Our data add to the previous two case reports suggesting that serum levels of angiogenic factors are similar to those reported for patients without kidney disease, except for PIGF levels, which are somewhat higher, consistent with data from Cornelis et al. In summary, we report a case of pregnancy in a woman who underwent intensive hemodialysis and had a relatively good outcome. Despite the presence of worsening hypertension, there was no evidence of PE. We suggest that monitoring of circulating angiogenic factors might be helpful to diagnose PE in this population. It is also essential to establish reference levels of these markers in pregnant patients on dialysis. Pregnancy on dialysis remains relatively rare and reliance on case reports such as ours will be required to guide the utilization of angiogenic factor measurements in dialysis patients.

## Consent

Written informed consent was obtained from the patient for publication of this Case report and any accompanying images.

## Ethics

Ethics approval was obtained from Ottawa Hospital Research Ethics Board and patient consented to the study.
